# Dune Morphology Classification and Dataset Construction Method Based on Unmanned Aerial Vehicle Orthoimagery

**DOI:** 10.3390/s24154974

**Published:** 2024-07-31

**Authors:** Ming Li, Zekun Yang, Jiehua Yan, Haoran Li, Wangzhong Ye

**Affiliations:** School of Energy and Transportation Engineering College, Inner Mongolia Agricultural University, Hohhot 010018, China; azk942982203@163.com (Z.Y.); yjh19990606@126.com (J.Y.); conan404@163.com (H.L.); pe_yewangzhong@126.com (W.Y.)

**Keywords:** UAV orthoimagery, dune classification, dataset, convolutional neural network, image classification

## Abstract

Dunes are the primary geomorphological type in deserts, and the distribution of dune morphologies is of significant importance for studying regional characteristics, formation mechanisms, and evolutionary processes. Traditional dune morphology classification methods rely on visual interpretation by humans, which is not only time-consuming and inefficient but also subjective in classification judgment. These issues have impeded the intelligent development of dune morphology classification. However, convolutional neural network (CNN) models exhibit robust feature representation capabilities for images and have achieved excellent results in image classification, providing a new method for studying dune morphology classification. Therefore, this paper summarizes five typical dune morphologies in the deserts of western Inner Mongolia, which can be used to define and describe most of the dune types in Chinese deserts. Subsequently, field surveys and the experimental collection of unmanned aerial vehicle (UAV) orthoimages for different dune types were conducted. Five different types of dune morphology datasets were constructed through manual segmentation, automatic rule segmentation, random screening, and data augmentation. Finally, the classification of dune morphologies and the exploration of dataset construction methods were conducted using the VGG16 and VGG19 CNN models. The classification results of dune morphologies were comprehensively analyzed using different evaluation metrics. The experimental results indicate that when the regular segmentation scale of UAV orthoimages is 1024 × 1024 pixels with an overlap of 100 pixels, the classification accuracy, precision, recall, and F1-Score of the VGG16 model reached 97.05%, 96.91%, 96.76%, and 96.82%, respectively. The method for constructing a dune morphology dataset from automatically segmented UAV orthoimages provides a reference value for the study of large-scale dune morphology classification.

## 1. Introduction

Desertification and land desertification are among the most significant ecological and environmental issues globally. In the inland northwest of China, they are primarily manifested as desertification. Dunes, as the main geomorphological type of desert, are influenced by both internal and external environmental factors such as sand sources, topography, vegetation, and moisture [1]. The formation process of dunes is complex, leading to the existence of various dune classification systems. These can be categorized into four main methods: dynamic classification, morphological classification, sedimentary condition classification, and comprehensive classification [2]. Morphological characteristics serve as the primary basis for delineating dune types and contain abundant information on the evolution of dune morphologies, providing crucial guidance for research on the prevention and control of desertification [3]. Exploring the classification of dune morphologies based on CNN advances the collection and processing of desert information in an intelligent direction, enhancing the efficiency and accuracy of desert information gathering and laying a solid foundation for the prevention and treatment of desertification. In the field of deep learning, which is driven by big data, the quantity and quality of the dataset are key factors affecting the performance of image classification models [4]. Currently, there is a gap in publicly available datasets that utilize UAV remote sensing technology to obtain images of dune morphologies. For dunes, which are large-scale, feature-complex, and similar research subjects, the construction of the dataset must consider whether the images include typical morphological characteristics of different dune types, whether they contain similar features across different types, and the efficiency of construction. Therefore, the study of manual segmentation through visual interpretation and automatic segmentation at various scales provides foundational data for the automatic classification of dune morphologies.

Due to the complex and diverse morphological characteristics of dunes, which often exhibit similarities, current classification methods predominantly rely on manual visual interpretation. With the advancement of artificial intelligence (AI) technology, integrating deep learning techniques with dune morphology classification can significantly enhance the efficiency and accuracy of the process. Cui [5] utilized multi-source remote sensing data and integrated it with the ResNet50 model to achieve the classification of the Gurbantunggut Desert. By combining the FCN–VGG model with object-oriented multi-scale segmentation, the classification accuracy at the boundaries of dunes was effectively improved. Tang et al. [6] used Landsat-8 remote sensing imagery as foundational data, employing the SandUnet model for dune detection, followed by a fine-tuned MobileNet model to classify dune morphologies in the Taklamakan and Sahara Deserts. Van der Merwe et al. [7] employed CNN models to identify barchan dunes on Earth. Azzaoui et al. [8] utilized high-resolution IKONOS satellite imagery as a data source and combined a clustering-based image segmentation approach with a transfer learning method based on the deep learning AlexNet model to achieve barchan sand dunes collision detection with an experimental accuracy of 82.01%. Cunez et al. [9] employed a CNN model, specifically YOLOv8, utilizing a dataset constructed from multi-source remote sensing imagery and various types of images. They conducted detection of individuals and groups of barchan dunes on Mars and Earth, achieving confidence scores (estimated accuracy for each detected object) within 70% and 90%, with an average precision mean reaching 99%. Current research often relies on satellite remote sensing imagery as the data source. While these data offer extensive coverage, the resolution and update frequency may not meet the needs of certain specific applications. For instance, the resolution of satellite imagery might be insufficient to capture small-scale changes in dune morphology, and the long update cycles are not conducive to real-time dynamic monitoring of dune morphology. Utilizing high-resolution orthoimagery obtained from UAVs can complement the shortcomings of satellite remote sensing data, enhancing the accuracy and timeliness of dune morphology classification.

The quality of the dataset is one of the critical factors influencing the performance of classification tasks. Given the large scale and subtle features of dune morphologies, which differ from the more distinct characteristics found in other domains such as precision agriculture, tree species, and plant classification, the creation of a dune dataset presents a unique challenge. Different types of datasets can exert varying influences on the classification models. Jiang et al. [10] used GF-2 satellite remote sensing imagery as a data source to construct forest stand-type datasets with four different image patch sizes. They compared the classification accuracy and effectiveness on CNN models, and the experimental results indicated that the classification performance was optimal with a 9 × 9 image patch, achieving an overall accuracy of 94.78% and a Kappa coefficient of 0.9318. Xu et al. [11] proposed a method for constructing an urban architectural style dataset, which resulted in a classification accuracy of 57.8%, a recall rate of 80.91%, and an F1-Score of 0.634. Kasimu et al. [12] explored the impact of the number of training samples in the dataset on model classification accuracy using UAV imagery of oasis plant communities in the heart of the desert as the data source, revealing that there is a certain dependency between the model’s classification accuracy and an appropriate number of training samples. In the aforementioned studies, the construction of the datasets primarily focused on tree species, buildings, and vegetation. There is a scarcity of research on the construction of dune morphology classification datasets based on UAV orthoimagery. Considering the characteristics of dune morphology, it is meaningful to study the construction methods for dune morphology classification datasets.

This paper proposes a method for constructing dune morphology classification datasets based on UAV orthoimagery. The contributions of this paper are summarized as follows: Firstly, the paper identifies and summarizes five typical dune morphological types found in the deserts of western Inner Mongolia, providing detailed descriptions of the characteristics of each dune morphology. These can be used to define and classify most dunes in China. Secondly, five different types of dune morphologies datasets are constructed by manual segmentation, automatic rule segmentation, random screening, and data augmentation using UAV orthoimagery as a data source. Lastly, the paper employs the VGG16 and VGG19 models to classify the datasets of the five different types of dune morphologies, exploring methods suitable for constructing datasets for dune morphology classification. This work holds significant value for the development of intelligent desert information collection.

## 2. Materials and Methods

### 2.1. Overview of the Study Area

**Figure 1:**
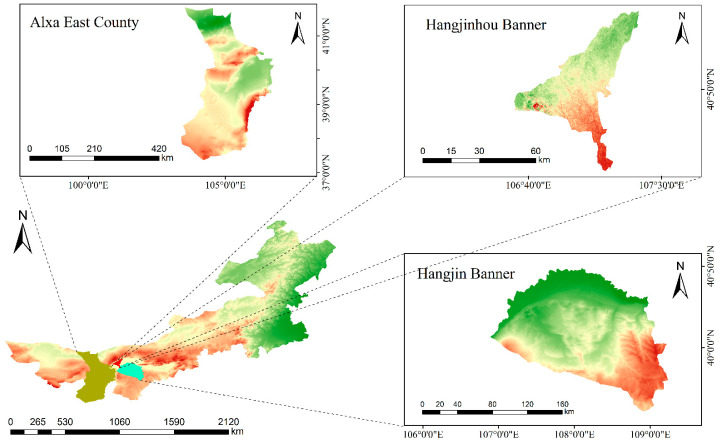
Study area map.

The present experiment focuses on the deserts of western Inner Mongolia as the study area, including the Ulan Buh Desert, Hobq Desert, and Yamaleike Desert. Field investigations have revealed the following: The Ulan Buh Desert experiences prevailing northerly winds in winter and southerly winds in summer, with typical dune morphologies including barchan dunes and dune chains, as well as reticulate dunes and nebkhas [13]. The UAV data collection was conducted in desert areas within Alxa East County, near Wuhai City, and within the borders of Hangjinhou Banner. The Hobq Desert is characterized by northwesterly winds in winter and southeasterly winds in summer, with the main dune morphologies being barchan dunes and dune chains, reticulate dunes, and nebkhas. The UAV data collection primarily targeted the desert area near Duguitara Town in Hangjin Banner. The Yamaleike Desert has a consistent southwesterly wind throughout the year, with a significant presence of isolated barchan dunes along the southeastern fringes of the desert [14]. The UAV data collection took place within the territory of Alxa East County. The specific geographic location is depicted in Figure 1.

### 2.2. Dune Morphology Classification System

Scholars both domestically and internationally have proposed a variety of different standards for dune classification. This study will classify the dunes of the western Inner Mongolia region based on the distribution types of dunes in the study area and Zheng Wu’s [15] principle of genesis morphology. A total of five dune morphology types have been summarized, which will serve as the foundation for the research on automatic dune morphology classification and dataset construction.

(1) Barchan dunes and dune chains exhibit a crescent-shaped planar morphology, influenced by unidirectional winds. These dunes have two wing-like extensions downwind, with asymmetrical slopes on either side. The windward slope is convex and relatively gentle, while the leeward slope is concave and steeper. Under conditions of abundant sand supply, a chain of barchan dunes, known as a dune chain, can form [16].
(2) Linear dunes are formed under the influence of two winds that intersect at an acute angle. Their prominent feature is a long and straight dune crest line, with symmetrical slip faces on either side of the dune. In deserts, linear dunes are often arranged in parallel and exhibit relatively regular spacing.
(3) Reticulate dunes are composed of two sets of intersecting dunes, and the plane shape is grid. Based on the differences in the morphological characteristics of reticulate dunes, they can be further divided into square reticulate dunes and long reticulate dunes [17].
(4) Nebkhas form when aeolian sand is obstructed by shrubby vegetation, causing a reduction in wind speed and the continuous accumulation of sand particles at the base of the shrubs. In plane view, these dunes typically exhibit an oval, circular, or tadpole-like shape, with a certain amount of shrubby vegetation covering the top of the dune mound [18].
(5) In flat sandy land, there are no dunes of any form, presenting an extensive and continuous planar morphology [19].

**Figure 2:**
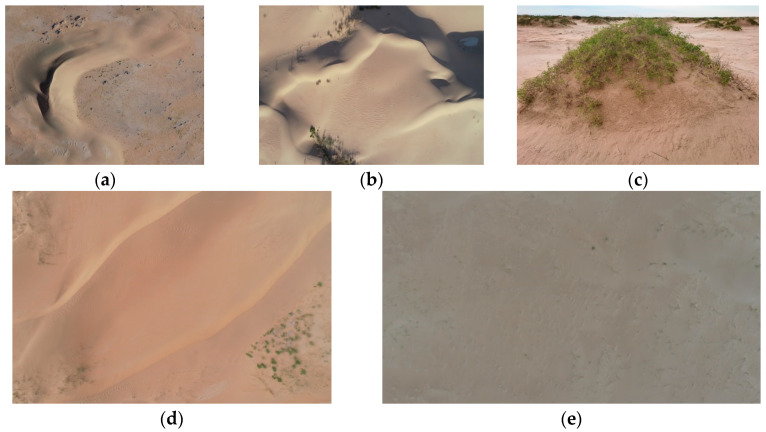
Schematic diagram of dune morphology types: (**a**) Barchan dunes and dune chains; (**b**) Reticulate dunes; (**c**) Nebkhas; (**d**) Linear dunes; (**e**) Flat sandy land.

Example images of each type are shown in Figure 2.

### 2.3. Data Sources

**Figure 3:**
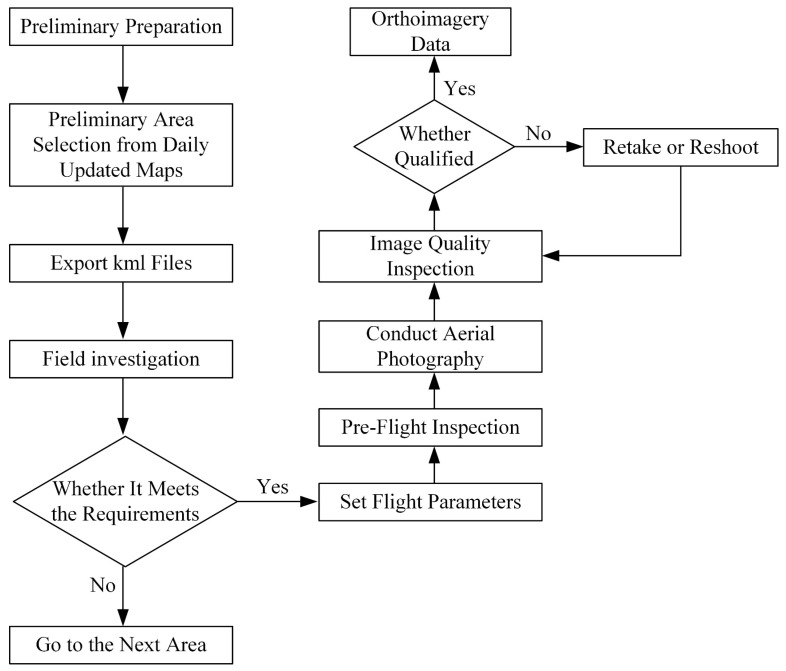
UAV data collection process.

The data utilized in the experiment consists of orthophotos acquired through low-altitude remote sensing by UAV. The specific UAV model employed is the DJI Phantom 4 Pro, equipped with a DJI FC6310 camera (DJI, Shenzhen, China). The relevant parameters are detailed in Table 1. The experimental data collection was conducted in two phases, with field surveys and UAV aerial photography carried out in different study areas in late April 2023 and early August 2023, respectively. The process of UAV imagery collection is depicted in Figure 3.

**Figure 4:**
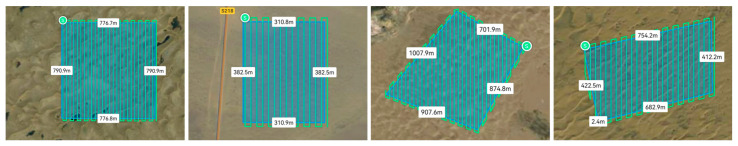
UAV flight paths; S represents the starting point for the UAV to take photographs.

Due to the differences in typical dune morphology types and distribution areas in each desert, to ensure data collection efficiency, preliminary selection of data collection areas was conducted through the daily updated maps on the Ovitalmap platform before the experiment. For each type of dune, 2–3 areas were planned, and kml files were exported to facilitate subsequent UAV data collection. Before UAV aerial photography, on-site manual visual interpretation of the collection area was required to assess whether it met the data collection requirements. UAV data collection was generally scheduled from 10:00 to 15:00 when the weather was clear and under natural light conditions. The flight mission involves route flying, with some UAV flight paths illustrated in Figure 4. Flight parameters related to different types of dunes are shown in Table 2. A total of 10,769 photos with a resolution of 5472 × 3078 pixels were collected.

### 2.4. Dataset Construction Method

**Figure 5:**
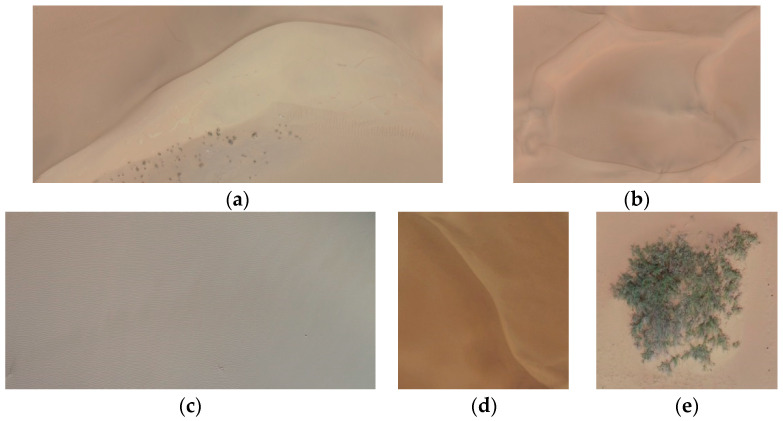
Manual segmentation dataset example diagram; (**a**) Barchan dunes and dune chains; (**b**) Reticulate dunes; (**c**) Flat sandy land; (**d**) Linear dunes; (**e**) Nebkhas.

A total of five different types of dune morphology datasets were constructed. A total of 1923 images were collected from the UAV data in April 2023, which were then manually segmented through visual interpretation. The image data predominantly consisted of individual, complete dune images with distinct morphological characteristics and varying pixel resolutions. Data augmentation techniques such as zooming, rotating, adjusting brightness, and adding different types of noise were applied to construct a manually segmented dune morphologies dataset containing 3788 images. This dataset was then randomly divided into a training set and a test set in an 8:2 ratio [20,21]. The quantities of each dune type within the training and test sets are detailed in Table 3, and schematic illustrations of the different dune types are presented in Figure 5.

**Figure 6:**
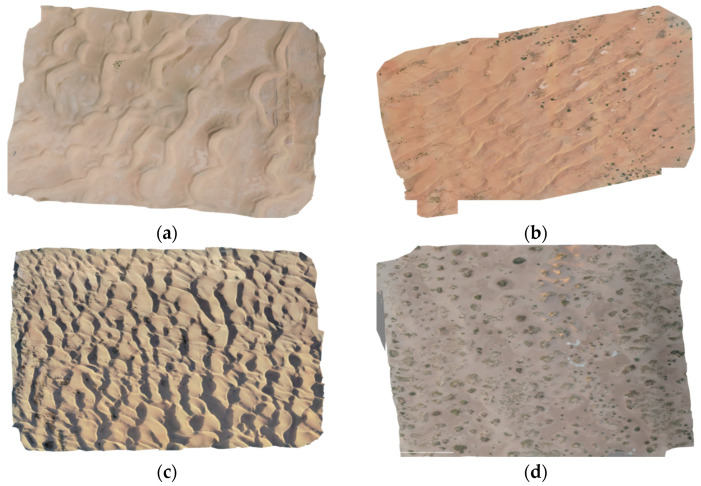
UAV orthoimagery; (**a**) barchan dunes and dune chains; (**b**) linear dunes; (**c**) reticulate dunes; (**d**) reticulate dunes.

The UAV data captured in early August 2023 was processed for orthoimage mosaicking, resulting in partial orthoimages as shown in Figure 6. Taking into account the typical dune morphologies of the western Inner Mongolia desert, including textural characteristics, color, and spatial distribution, the UAV orthoimages were systematically auto-segmented into regular blocks of 1024 × 1024 pixels, 512 × 512 pixels, 256 × 256 pixels, and 128 × 128 pixels. The specific method for constructing the dataset is as follows.

The UAV orthoimages were systematically segmented into regular blocks of 1024 × 1024 pixels with an overlap of 100 pixels. To ensure the dataset’s balance, diversity, and representativeness, data augmentation techniques were employed, resulting in a total of 14,609 image data points. These were then randomly divided into training and testing sets in an 8:2 ratio, with specific quantities detailed in Table 4.

The UAV orthoimageries were automatically segmented into a dataset with 512 × 512 pixel blocks, utilizing an overlap of 100 pixels. Data augmentation was applied to various dune types, including flat sandy land, nebkhas, and barchan dunes and dune chains. This process resulted in a dataset comprising 47,354 image data. The dataset was then randomly divided into training and testing sets in an 8:2 ratio, with the specific quantities outlined in Table 5.

For the dataset consisting of 256 × 256 pixels blocks, an overlap of 100 pixels was set. From the UAV orthoimagery of reticulate dunes, which was 43,082 × 34,407 pixels in size, segmentation and filtering resulted in 53,915 image data. After random screening at a ratio of 0.2, the dataset was reduced to 10,782 images. For the linear dunes, which were captured in UAV orthoimages sized 39,573 × 33,417 pixels, segmentation and filtering yielded 38,608 images. Applying a random sampling ratio of 0.3, the number of images was further refined to 11,582. Flat sandy land, after data augmentation, contributed an additional 9670 images to the dataset. In total, the dataset comprised 58,798 images, which were then randomly divided into training and testing sets in an 8:2 ratio, with the specific quantities detailed in Table 6.

**Figure 7:**
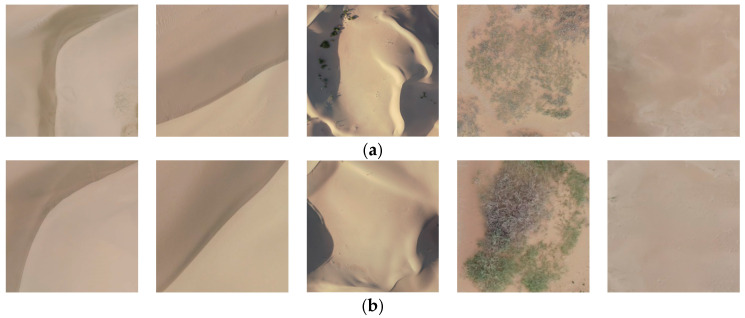

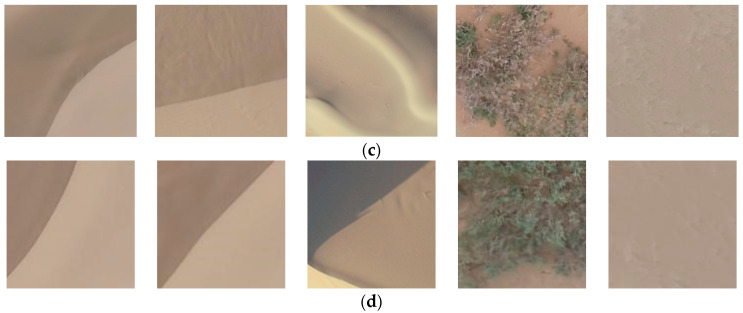
Sample image of different scale segmentation datasets: (**a**) 1024 × 1024 pixels dataset; (**b**) 512 × 512 pixels dataset; (**c**) 256 × 256 pixels dataset; (**d**) 128 × 128 pixels dataset.

The dataset consisting of 128 × 128 pixels blocks was created with an overlap setting of 10 pixels. Orthoimages of reticulate dunes, barchan dunes, and dune chains captured by UAV were segmented, and then a random selection of data was performed at a ratio of 0.2. Data augmentation was applied to the flat sandy land. This process resulted in a total of 61,624 image data. The dataset was subsequently divided into training and testing sets in an 8:2 ratio, with the specific quantities presented in Table 7. Example images of the datasets with four different segmentation scales are illustrated in Figure 7.

### 2.5. CNN Model Selection and Parameter Configuration

CNN is a class of feedforward neural networks that automatically learn representations of raw data through multiple convolutional and pooling layers, thereby exhibiting substantial feature extraction capabilities. CNN has found broad application in tasks related to image classification due to its proficiency in capturing and learning spatial patterns within images. The VGG16 and VGG19 models are selected for their strong transfer learning capabilities.

**Figure 8:**
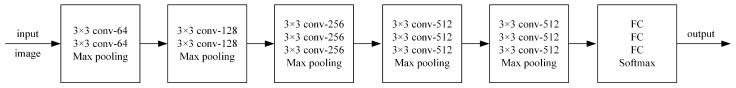
VGG16 network structure diagram.

VGG16 refers to a CNN architecture that comprises a total of 16 layers, including 13 convolutional layers and 3 fully connected layers. The convolutional layers are organized into 5 blocks, each utilizing a series of small, 3 × 3 convolutional kernels. The Rectified Linear Unit (ReLU) activation function is employed, as expressed by Equation (1), to enable the representation of nonlinear data. Following each convolutional block is a pooling layer with a 2 × 2 kernel, which employs max pooling to downsample the spatial dimensions of the feature maps [22]. The fully connected layers serve as the “classifier” within the model, interpreting the high-level features extracted by the convolutional layers to perform the final classification. The network architecture of VGG16 is illustrated in Figure 8.

(1) f(x)=max(0,x)

**Figure 9:**
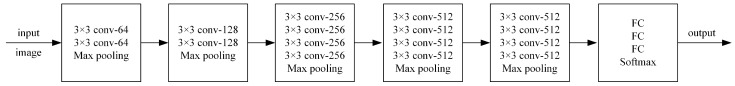
VGG19 network structure diagram.

VGG19 and VGG16 are fundamentally similar in architecture, with the primary difference being the depth of the network. VGG19 consists of 19 layers in total, including 16 convolutional layers and 3 fully connected layers, organized into 5 convolutional blocks. The additional convolutional layers in VGG19 allow for further feature extraction from the images, enhancing the network’s expressive power. The specific network architecture of VGG19 is depicted in Figure 9.

The network structure is constructed, and the models are trained using the deep learning framework PyTorch, with the VGG16 and VGG19 models as the foundation. Pre-training is performed on the large-scale dataset ImageNet, and the parameters of the last fully connected layer are fine-tuned using the Adam optimizer, while the remaining layers serve as fixed feature extractors for the dune morphologies datasets. The loss function selected is the cross-entropy loss function, with an initial learning rate of 0.01. The learning rate is adjusted using the stepLR strategy, where the learning rate is reduced to 0.5 of its original value after every 5 epochs. The experiment is run for 30 epochs, with a batch size of 64. The experimental environment configuration is detailed in Table 8.

### 2.6. Model Evaluation Metrics

To quantitatively evaluate the performance of the models, we have selected five metrics: the confusion matrix, accuracy, precision, recall, and F1-Score. The confusion matrix is presented in Table 9, while the formulas for accuracy, precision, recall, and F1-Score are shown in Equations (2)–(5) [23].

(2) $$Accuracy=\frac{TP+TN}{TP+TN+FP+FN}$$

(3) $$Precision=\frac{TP}{TP+FP}$$

(4) $$Recall=\frac{TP}{TP+FN}$$

(5) $$F1-Score=\frac{2\times Precision\times Recall}{Precision\times Recall}$$

## 3. Results

### 3.1. Single Category Classification Results and Analysis

**Figure 10:**
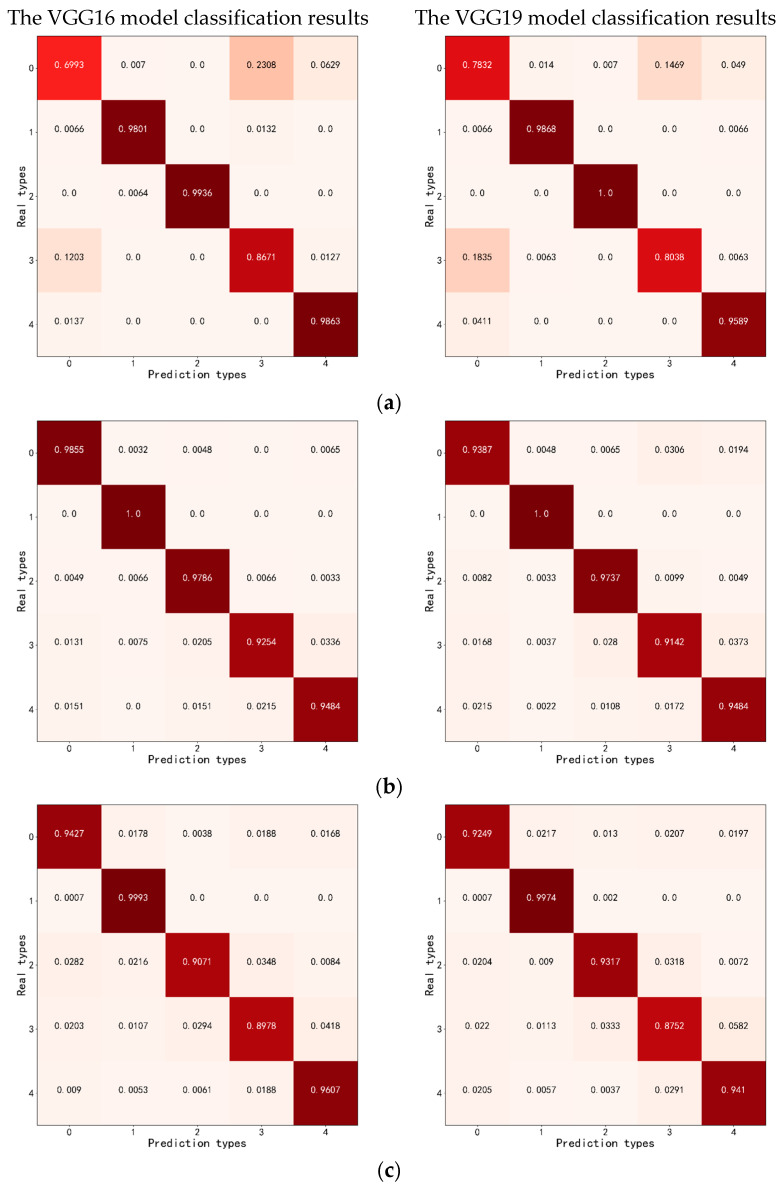

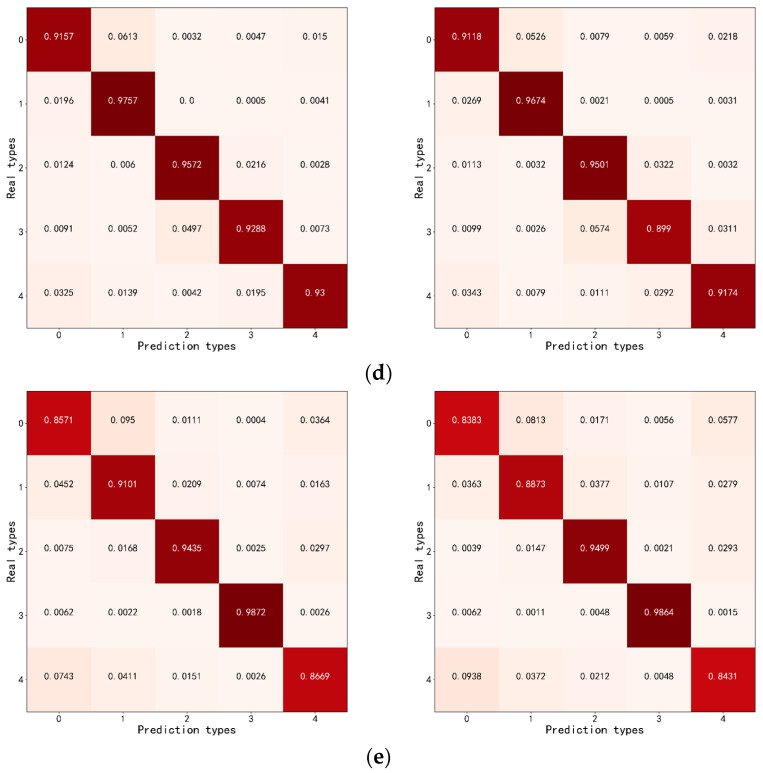
Confusion matrices normalization results. In the figure: 0 represents barchan dunes and dune chains; 1 represents flat sandy land; 2 represents nebkhas; 3 represents linear dunes; 4 represents reticulate dunes; (**a**) manually segmented dataset; (**b**) 1024 × 1024 pixels dataset; (**c**) 512 × 512 pixels dataset; (**d**) 256 × 256 pixels dataset; (**e**) 128 × 128 pixels dataset.

Figure 10 presents the normalized results of the confusion matrices for the test sets using different dataset construction methods. It can be observed from the figure that in the datasets of 1024 × 1024 pixels, 512 × 512 pixels, and 256 × 256 pixels, the flat sandy land has the highest classification accuracy. Compared with other types of dunes, flat sandy land lacks the undulations characteristic of dunes, resulting in more distinct and recognizable features.

(a) In the manually segmented dataset, the nebkhas exhibit the highest classification accuracy. This is attributed to the presence of shrubby vegetation covering the surface of these dunes, which provides a distinct textural characteristic that sets them apart from other types of dunes. Conversely, barchan dunes and dune chains are most frequently misclassified. This misclassification is likely due to their similar morphological features when compared with linear dunes, making it challenging to differentiate them based on visual or textural cues alone.
(b) In the 1024 × 1024 pixels dataset, the VGG16 model often misclassifies linear dunes as nebkhas. This misclassification can be attributed to the presence of shrubby vegetation in the areas where linear dunes were photographed, which may lead to confusion during the classification process. The VGG19 model, on the other hand, predominantly misclassifies barchan dunes and dune chains as linear dunes and reticulate dunes. Additionally, there was a significant amount of misclassification between linear dunes and reticulate dunes, which resulted in lower classification accuracy rates for barchan dunes, dune chains, and reticulate dunes.
(c) In the 512 × 512 pixels dataset, the VGG16 model frequently misclassifies barchan dunes and dune chains as linear dunes and flat sandy land. For the VGG19 model, linear dunes were most often misclassified as reticulate dunes. This is likely because, during the small-scale regular segmentation, the reticulate dunes may only include a portion of their morphological features, which can resemble those of linear dunes, thus making misclassifications more likely.
(d) In the 256 × 256 pixels dataset, barchan dunes and dune chains were often misclassified as flat sandy land. When segmenting the orthoimages of barchan dunes and dune chains, the interdune areas share similar characteristics with flat sandy land. At the smaller segmentation scale, a single image may not capture the morphological features of barchan dunes and dune chains, leading to a higher likelihood of misclassification between these two types.
(e) In the 128 × 128 pixels dataset, due to the small segmentation scale, there was a significant amount of misclassification. Specifically, barchan dunes and dune chains were frequently misclassified as flat, sandy land. Additionally, reticulate dunes were also misclassified, particularly barchan dunes and dune chains, as some of their morphological features are similar. The smaller segmentation scale does not fully capture the morphological characteristics, which can lead to confusion when the CNN models are extracting deep semantic features.

### 3.2. Results and Analysis of Different Dataset Construction Methods

The classification results for datasets of different dune morphologies are presented in Table 10.

(a) When the dataset is segmented at a scale of 1024 × 1024 pixels using a regular segmentation method, the VGG16 model achieves the highest accuracy, precision, recall, and F1-Score, which are 97.05%, 96.91%, 96.76%, and 96.82%, respectively.
(b) The VGG16 model’s classification accuracy, arranged from highest to lowest, is as follows: the dataset with 1024 × 1024 pixels dataset, the 512 × 512 pixels dataset, the 256 × 256 pixels dataset, the 128 × 128 pixels dataset, and the manually segmented dataset.
(c) The VGG19 model’s classification accuracy, arranged from highest to lowest, is as follows: the 1024 × 1024 pixels dataset, the 512 × 512 pixels dataset, the 256 × 256 pixels dataset, the manually segmented dataset, and the 128 × 128 pixels dataset.

Comprehensive analysis indicates that CNN models demonstrate superior performance when classifying dune morphology datasets with a segmentation scale of 1024 × 1024 pixels and an overlap of 100 pixels. Compared with manually segmented datasets, automatically segmented datasets offer higher efficiency.

### 3.3. Visualization and Analysis of Semantic Feature Results for Test Set Images

**Figure 11:**
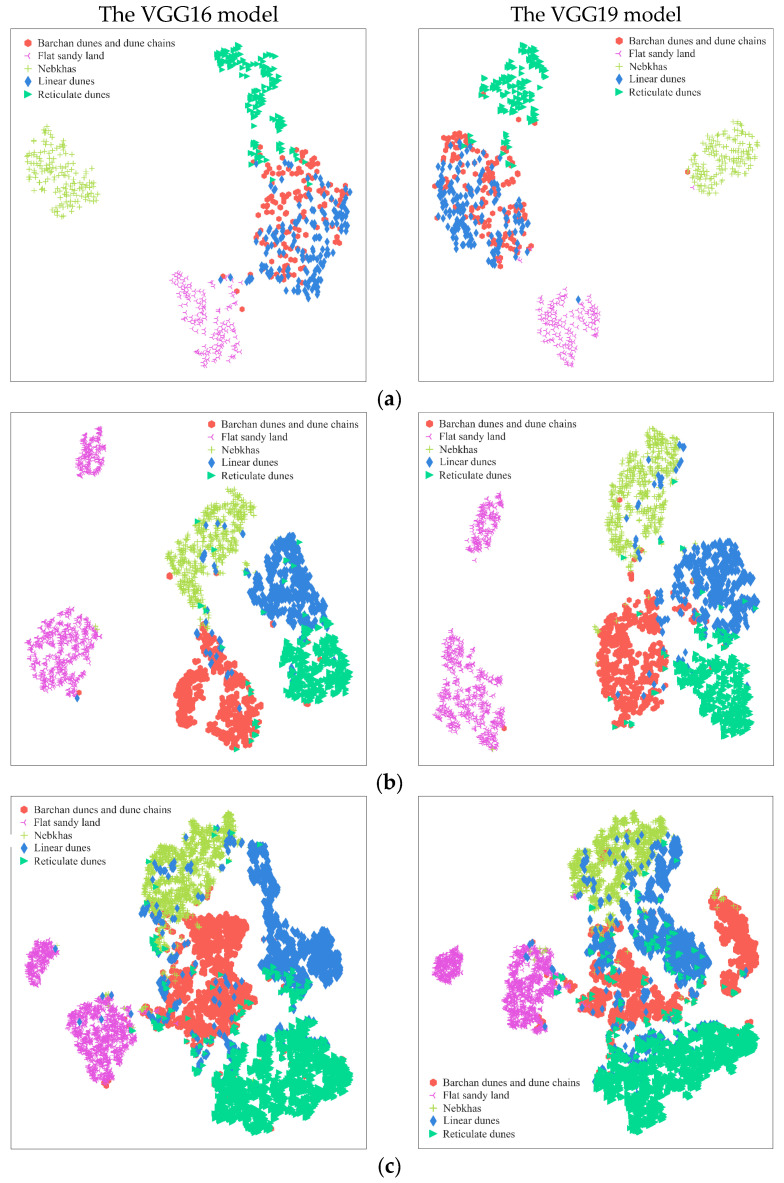

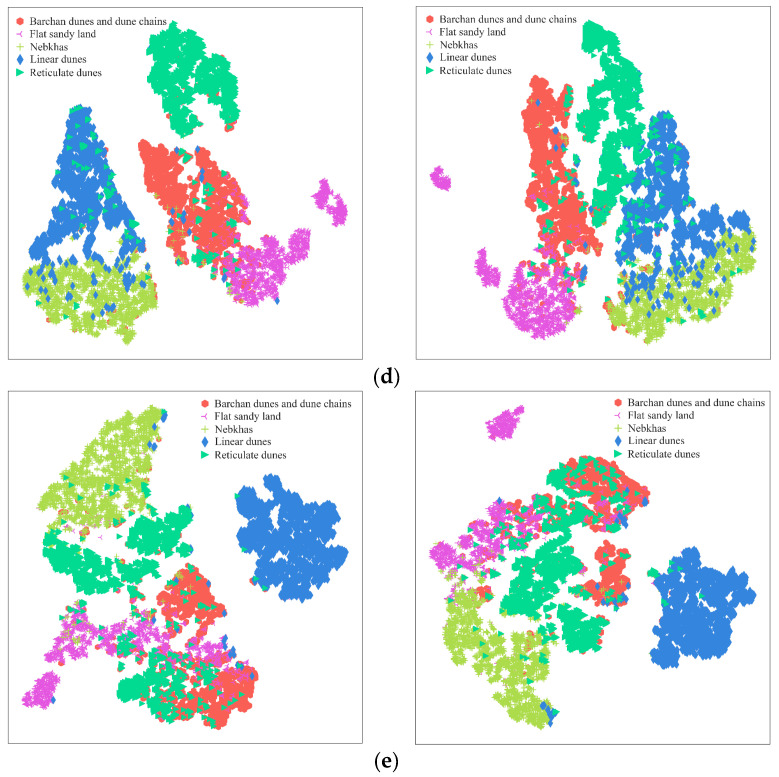
Semantic feature map; (**a**) manually segmented dataset; (**b**) 1024 × 1024 pixels dataset; (**c**) 512 × 512 pixels dataset; (**d**) 256 × 256 pixels dataset; (**e**) 128 × 128 pixels dataset.

To intuitively analyze the semantic distances between different dune morphology types, t-SNE dimensionality reduction visualization was employed. The outputs from the last fully connected layer of the VGG16 and VGG19 models were used as semantic features. As shown in Figure 11, which illustrates the semantic feature maps for classifications across different datasets, the following observations can be made:

(a) In the manually segmented dataset, nebkhas, flat sandy land, and reticulate dunes exhibit distinct cluster structures. However, there is an overlap in the data points for barchan dunes, dune chains, and linear dunes, suggesting that these dune types have closer semantic distances and share similar textural characteristics.
(b) In the 1024 × 1024 pixels dataset, each type exhibits a distinct cluster structure, while barchan dunes and dune chains, linear dunes, reticulate dunes, and nebkhas display close semantic distances.
(c) In the 512 × 512 pixels dataset, data points for barchan dunes and dune chains, nebkhas, and linear dunes are interspersed, with the three exhibiting similar textural characteristics.
(d) In the 256 × 256 pixels dataset, the classification performance of the VGG16 model surpasses that of the VGG19 model. The VGG16 model exhibits distinct cluster structures, whereas in the VGG19 model, there is a clear misclassification across various types, with data points showing a pronounced interspersion.
(e) In the 128 × 128 pixels dataset, data points for nebkhas and linear dunes are clustered together, while other types are interspersed, exhibiting close semantic distances and similar morphological features.

Semantic feature maps can intuitively interpret the results of single-category classification. The morphologies of dunes across different types exhibit similarities, which can lead to misclassification by CNN models, thereby reducing accuracy. In comparison, the 1024 × 1024 pixels dataset has a distinct cluster structure that stands out from other datasets, demonstrating that the optimal classification results for dune morphologies are achieved when the segmentation scale is set to 1024 × 1024 pixels. This segmentation scale is suitable for the construction of dune morphology datasets.

## 4. Discussion

### 4.1. Methods for Dune Morphology Dataset Construction

The present study proposes a method for constructing dune morphology classification datasets suitable for CNN models based on orthoimagery acquired by UAVs. UAV imagery provides a more detailed observation of the ground and is capable of capturing subtle changes and detailed features of dune morphology, which is crucial for enhancing the accuracy of dune classification. Research by Yang et al. [24] has demonstrated the effectiveness of UAV imagery combined with multi-scale segmentation methods in forestry resource surveys, indicating the potential of UAV imagery in extracting features of complex terrains. Similarly, studies by Yang et al. [25] have showcased the application of UAV imagery in conjunction with deep learning models in the field of precision agriculture, further confirming the generalizability of combining UAV imagery with deep learning.

The production of classification datasets is a time-consuming and labor-intensive task. In this experiment, a total of five different types of dune morphology datasets were constructed, including a manual segmentation dataset through visual interpretation and four datasets segmented at different scales using rule-based methods. Image data at different segmentation scales encompasses distinct dune morphologies and features. Larger segmentation scales provide more complete representations of dune morphologies but result in fewer segmented images, leading to datasets with more pronounced sample features. Conversely, smaller segmentation scales capture partial, typical features of dune morphologies. Due to the similarity among various dune morphological features, misclassification may occur. However, this approach yields a greater number of segmented images, enriching the dataset with a more diverse set of samples. Taking into account factors such as the textural characteristics, color, spatial distribution of dune morphologies, and UAV flight parameters within the study area, four different segmentation scales—1024 × 1024 pixels, 512 × 512 pixels, 256 × 256 pixels, and 128 × 128 pixels—were selected to perform rule-based segmentation of UAV orthoimagery. The experimental results indicate that the dataset constructed at a segmentation scale of 1024 × 1024 pixels demonstrates more pronounced cluster structures and better classification results. Due to the fixed scale of orthoimagery, when a larger segmentation scale (such as 2048 × 2048 pixels) is used, the resulting number of images is reduced, but each image contains more feature information. Consequently, this can lead to insufficient feature learning by the models, which may affect the classification effectiveness of dune morphologies. As shown in Table 11, with the increase in segmentation scale, the model training time becomes longer. This is because larger segmentation scales encompass more feature information, which in turn increases the learning time for the model. The classification model trained on the 1024 × 1024 pixels dataset has already reached a training time of nearly 27 h, with a classification accuracy of 97.05%. Considering the trade-offs, if the segmentation scale is larger, the required time increases, making the cost-effectiveness of constructing dune morphology datasets less favorable.

Zhao [1] selected three different segmentation scales of 20 × 20 pixels, 40 × 40 pixels, and 60 × 60 pixels for regular slicing of remote sensing imagery. The results indicated that the ResNet50 model achieved the highest classification accuracy at a scale of 60 × 60 pixels, while the VGG19 model demonstrated the highest classification accuracy at a scale of 20 × 20 pixels. Jiang et al. [10] segmented remote sensing imagery into image patches of sizes 5 × 5, 7 × 7, 9 × 9, and 11 × 11. The experimental results showed that the classification effect was optimal with an image patch size of 9 × 9. In the aforementioned studies, the data sources are all satellite remote sensing images with a spatial resolution at the meter level; hence, their segmentation scales are smaller than the segmentation scales used in this experiment. Therefore, the research on constructing datasets based on different segmentation scales of UAV orthoimagery is both meaningful and well-founded.

### 4.2. Performance of Different Models in Dune Morphology Classification Datasets

A comparative analysis was conducted using the VGG16 and VGG19 models within the CNN on five different dune morphology classification datasets. Experimental results indicate that both the VGG16 and VGG19 models performed excellently on the 1024 × 1024 pixels dataset, achieving classification accuracies of 97.05% and 95.75%, respectively. The analysis suggests that larger segmentation scales can more completely reflect dune morphologies. For large-scale research subjects like dunes, the integrity of textural and morphological features positively influences model classification. The segmentation scale of 1024 × 1024 pixels captures more information about dune morphologies compared with the other three scales, allowing the dataset to more accurately delineate the characteristic information between different dunes. At other smaller segmentation scales, the representation of dune morphologies and textural features is insufficient, which affects the classification accuracy of CNN models. Rule-based segmented datasets, as opposed to manually segmented datasets, contain a larger number of images, and the morphological features learned during training are more pronounced; hence, the classification accuracy of manually segmented datasets is lower. Lin et al. [26] conducted a study using UAV imagery in conjunction with the DenseNet model for tree species classification, achieving a maximum accuracy of 87.54%. Pouliot et al. [27] explored the feasibility of using CNN on Landsat remote sensing imagery for wetland classification, with a classification accuracy of 68%. Dyrmann et al. [28] applied CNN to identify plant species in complex environments, reaching a classification accuracy of 86.2%. The classification results of this experiment are superior to those of the aforementioned studies; thus, the dune morphology classification dataset construction method and the selection of the CNN model in this paper are of reference value.

## 5. Conclusions

This paper uses UAV orthoimagery as the data source. It constructs five types of typical dune morphology datasets from the western desert of Inner Mongolia through manual segmentation and automatic rule-based segmentation. The study explores the classification of dune morphologies and the construction methods of the datasets using the VGG16 and VGG19 models within CNNs.

(1) When the segmentation scale of UAV orthoimagery is set to 1024 × 1024 pixels with an overlap of 100 pixels, the classification outcome for dune morphologies is optimal. The VGG16 model achieved classification accuracy, precision, recall, and an F1-Score of 97.05%, 96.91%, 96.76%, and 96.82%, respectively. Compared with the manually segmented dataset, these metrics improved by 6.32%, 6.27%, 6.23%, and 6.38%, respectively.
(2) The semantic feature maps of the test set visually demonstrate that the 1024 × 1024 pixels dataset has distinct cluster structures for each type of dune morphology, resulting in the best classification performance.

The deficiency and prospect of this study:

(1) Due to the significant differences in scale and height of the dunes in the study area, different Ground Sampling Distance (GSD) were utilized when collecting UAV data. The impact of varying GSD on the classification results of dune morphology can be further explored in future research.
(2) In future research, a larger collection of UAV orthoimagery data will be gathered to explore further the specific impact of varying quantities of each dune morphology type within the dataset on model classification. Additionally, the influence of different quantities of dune morphology datasets on model classification will be investigated.

## Figures and Tables

**Table 1:** UAV and camera-related parameters.

UAV Parameters	Numerical Value	Camera Parameters	Numerical Value/Form
Weight (including battery and paddles)	1388 g	Image sensor	1-inch CMOS effective pixels 20 million
Wheelbase	350 mm		
Maximum flight time	About 30 min	Lens	FOV84°8.8 mm/24 mm (35 mm format equivalent) f/2.8–f/11 with autofocus (Focus distance 1 m- infinity)
Maximum horizontal flight speed	72 km/h		
Maximum tilt angle	42°	Photo size	3:2 aspect ratio: 5472 × 3648 4:3 aspect ratio: 4864 × 3648 16:9 aspect ratio: 5472 × 3078
Maximum bearable wind speed	10 m/s		
Maximum takeoff altitude	6000 m		
Satellite Positioning Module (SPM)	GPS/GLONASS dual mode	Picture format	JPE; GDNG(RAW), JPEG + DNG

**Table 2:** UAV flight parameters.

Dune Types	Flight Altitude/m	Lateral Overlap	Forward Overlap	GSD/cm/Pixels
Linear dunes	80	75%	80%	2.19
Nebkhas	30	75%	80%	0.82
Barchan dunes and dune chains	80	75%	80%	2.19
Reticulate dunes	100	75%	80%	2.74
Flat sandy land	80	70%	80%	2.19

**Table 3:** Manual segmentation of dune morphology datasets.

Dune Types	Number of Training Sets	Number of Test Sets	Total
Flat sandy land	606	151	757
Barchan dunes and dune chains	575	143	718
Reticulate dunes	585	146	731
Nebkhas	635	157	792
Linear dunes	632	158	790

**Table 4:** 1024 × 1024 pixels dataset.

Dune Types	Number of Training Sets	Number of Test Sets	Total
Flat sandy land	2765	691	3456
Barchan dunes and dune chains	2480	620	3100
Reticulate dunes	1863	465	2328
Nebkhas	2436	608	3044
Linear dunes	2145	536	2681

**Table 5:** 512 × 512 pixels dataset.

Dune Types	Number of Training Sets	Number of Test Sets	Total
Flat sandy land	6042	1510	7552
Barchan dunes and dune chains	8314	2078	10,392
Reticulate dunes	9770	2442	12,212
Nebkhas	6672	1668	8340
Linear dunes	7087	1771	8858

**Table 6:** 256 × 256 pixels dataset.

Dune Types	Number of Training Sets	Number of Test Sets	Total
Flat sandy land	7736	1934	9670
Barchan dunes and dune chains	10,112	2527	12,639
Reticulate dunes	8627	2156	10,783
Nebkhas	11,300	2824	14,124
Linear dunes	9266	2316	11,582

**Table 7:** 128 × 128 pixels dataset.

Dune Types	Number of Training Sets	Number of Test Sets	Total
Flat sandy land	8592	2148	10,740
Barchan dunes and dune chains	9352	2338	11,690
Reticulate dunes	9259	2314	11,573
Nebkhas	11,191	2797	13,988
Linear dunes	10,907	2726	13,633

**Table 8:** Experimental environment configuration.

Name	Configuration
Operating system	Windows 10 64-bit operating system
Processor	Intel(R) Xeon(R) Silver 4216 CPU@2.10 GHz 2.10 GHz (2 Processors) (Intel, Santa Clara, CA, USA)
Video memory	NVIDIA GeForce RTX 3060 (NVIDIA, Santa Clara, CA, USA)
Memory	64 GB
Deep learning framework	pytorch2.0.1
Programming language	python3.9
CUDA version	12.2

**Table 9:** Confusion matrix.

Confusion Matrix		Predicted Value	
		Positive	Negative
True Value	Positive	True Positive = TP	False Negative = FN
	Negative	False Positive = FP	True Negative = TN

**Table 10:** Different dataset classification results.

Datasets	Models	Accuracy	Precision	Recall	F1-Score
Manually segmented dataset	VGG16	90.73%	90.64%	90.53%	90.44%
	VGG19	90.73%	90.57%	90.65%	90.59%
1024 × 1024 pixels dataset	VGG16	97.05%	96.91%	96.76%	96.82%
	VGG19	95.75%	95.47%	95.50%	95.47%
512 × 512 pixels dataset	VGG16	94.18%	94.09%	94.16%	94.09%
	VGG19	93.25%	93.21%	93.40%	93.28%
256 × 256 pixels dataset	VGG16	94.07%	94.00%	94.15%	94.03%
	VGG19	92.86%	92.80%	92.91%	92.83%
128 × 128 pixels dataset	VGG16	91.66%	91.24%	91.30%	91.23%
	VGG19	90.58%	90.09%	90.09%	90.08%

**Table 11:** Training time for different datasets.

Datasets	Classification Model	Training Time
1024 × 1024 pixels dataset	VGG16	26 h 30 min
	VGG19	26 h 56 min
512 × 512 pixels dataset	VGG16	18 h 26 min
	VGG19	19 h
256 × 256 pixels dataset	VGG16	7 h 16 min
	VGG19	11 h 3 min
128 × 128 pixels dataset	VGG16	6 h 47 min
	VGG19	8 h 3 min

## Data Availability

The datasets presented in this article are not readily available because the data are part of an ongoing study. The rest of the relevant data are within the article.
